# Systemic priming and intranasal booster with a BcfA-adjuvanted acellular pertussis vaccine generates CD4+ IL-17+ nasal tissue resident T cells and reduces *B. pertussis* nasal colonization

**DOI:** 10.3389/fimmu.2023.1181876

**Published:** 2023-05-18

**Authors:** Kacy S. Yount, Jesse M. Hall, Kyle Caution, Mohamed M. Shamseldin, Myra Guo, Keirsten Marion, Audra R. Fullen, Yimin Huang, Jennifer A. Maynard, Sally A. Quataert, Rajendar Deora, Purnima Dubey

**Affiliations:** ^1^ Department of Microbial Infection and Immunity, The Ohio State University, Columbus, OH, United States; ^2^ Department of Chemical Engineering, University of Texas-Austin, Austin, TX, United States; ^3^ Respiratory Pathogens Research Center, University of Rochester Medical Center, Rochester, NY, United States; ^4^ Department of Microbiology, The Ohio State University, Columbus, OH, United States

**Keywords:** *B. pertussis*, mucosal immunity, tissue-resident memory T cells, BcfA, pertussis vaccines, adjuvants

## Abstract

**Introduction:**

Resurgence of pertussis, caused by Bordetella pertussis, necessitates novel vaccines and vaccination strategies to combat this disease. Alum-adjuvanted acellular pertussis vaccines (aPV) delivered intramuscularly reduce bacterial numbers in the lungs of immunized animals and humans, but do not reduce nasal colonization. Thus, aPV-immunized individuals are sources of community transmission. We showed previously that modification of a commercial aPV (Boostrix) by addition of the Th1/17 polarizing adjuvant Bordetella Colonization Factor A (BcfA) attenuated Th2 responses elicited by alum and accelerated clearance of B. pertussis from mouse lungs. Here we tested whether a heterologous immunization strategy with systemic priming and mucosal booster (prime-pull) would reduce nasal colonization.

**Methods:**

Adult male and female mice were immunized intramuscularly (i.m.) with aPV or aPV/BcfA and boosted either i.m. or intranasally (i.n.) with the same formulation. Tissue-resident memory (TRM) responses in the respiratory tract were quantified by flow cytometry, and mucosal and systemic antibodies were quantified by ELISA. Immunized and naïve mice were challenged i.n. with Bordetella pertussis and bacterial load in the nose and lungs enumerated at days 1-14 post-challenge.

**Results:**

We show that prime-pull immunization with Boostrix plus BcfA (aPV/BcfA) generated IFNγ+ and IL-17+ CD4+ lung resident memory T cells (TRM), and CD4+IL-17+ TRM in the nose. In contrast, aPV alone delivered by the same route generated IL-5+ CD4+ resident memory T cells in the lungs and nose. Importantly, nasal colonization was only reduced in mice immunized with aPV/BcfA by the prime-pull regimen.

**Conclusions:**

These results suggest that TH17 polarized TRM generated by aPV/BcfA may reduce nasal colonization thereby preventing pertussis transmission and subsequent resurgence.

## Introduction

The Gram-negative obligate human pathogen *Bordetella pertussis* is the primary cause of whooping cough, or pertussis. Whole cell pertussis vaccines (wPV), used in the United States between the 1940s-1990s, provided long-lived immunity against infection in humans and effectively cleared the bacteria from the entire respiratory tract of mice and non-human primates (1–3). However, reactogenicity and public concerns regarding vaccine side effects led to the replacement of wPV in the 1990s with 3-5 component subunit vaccines adjuvanted with alum (4, 5). Although these acellular pertussis vaccines (aPV) prevent severe disease, they do not prevent establishment of a nasopharyngeal reservoir and subsequent transmission to vulnerable populations (6). Therefore, despite high global vaccine coverage of 85-100%, this disease remains endemic worldwide (7) and multiple recent outbreaks have resulted in significant morbidity and mortality in infants (8–12). While the disease is most severe and sometimes fatal in infants, in recent years there has been a shift in disease and infection burden to adolescents and young adults (13). Globally, estimates suggest that 15% of prolonged cough illnesses are due to *B. pertussis* infections with a yearly infection rate of 6% (14). However, symptomatic disease is not the only means of bacterial transmission (15). Experimental and computational studies indicate that children and adults vaccinated with aPV are asymptomatic transmitters of disease (16–20). A single primary case of pertussis can cause 12–17 new cases in susceptible individuals. This level of contagion is estimated to be similar to or higher than that of measles, influenza, mumps, rubella, polio, and smallpox (21). In 2015, pertussis and *B. pertussis* were included in the NIAID emerging infectious diseases and pathogens lists (22), respectively, emphasizing the need for more effective pertussis vaccines to improve global health.

Alum-adjuvanted aPV delivered intramuscularly (i.m.) elicits Th1/Th2 polarized systemic responses and fails to generate mucosal immunity (1). Furthermore, recent studies in mice show that i.m. aPV immunization exacerbates nasal colonization (2, 23). In contrast, natural infection and wPV immunization elicit Th1/17 polarized systemic responses (24) and elicit CD4+ T_RM_ and mucosal antibodies upon challenge with *B. pertussis* (1). These differences are two potential explanations for the suboptimal protection provided by aPV. Recent studies show that experimental subunit and whole-cell vaccines delivered intranasally (i.n.) elicit IL-17-producing CD4+ resident memory T cells (T_RM_) and mucosal antibodies that clear the infection from the entire murine respiratory tract (25–27), suggesting that inclusion of a Th1/17 polarizing adjuvant and mucosal vaccine delivery will improve aPV-mediated protection.

Bordetella Colonization Factor A (BcfA) is an outer membrane protein from the animal pathogen *Bordetella bronchiseptica* (28, 29). It is a pseudogene in the human pathogen *B. pertussis* (29). We showed that BcfA is an adjuvant that elicits Th1/17 polarized T cell and antibody responses to a variety of protein antigens (30, 31). Additionally, when combined with alum, BcfA attenuated alum primed Th2 responses. We further showed that i.m. priming and booster immunization of mice with a commercial aPV, Boostrix, with the addition of BcfA (aPV/BcfA) accelerated clearance of *B. pertussis* from mouse lungs.

Here we tested the hypothesis that a prime-pull immunization regimen with i.m. priming and i.n. booster with aPV/BcfA would generate mucosal immune responses and prevent the colonization of *B. pertussis* in both the upper and lower respiratory tracts of mice. We used the booster aPV, Boostrix, as the test vaccine because adolescents and adults who receive this booster are at risk for nasal colonization and transmission. Thus, modification of this formulation has potential clinical utility. While i.m. prime and boost with aPV/BcfA only cleared the lungs of mice, i.m. priming followed by i.n. booster of the same formula generated CD4+IL-17+ T_RM_ and significantly reduced *B. pertussis* colonization of the nose. Together, our data show that aPV/BcfA delivered by a prime-pull immunization regimen generates immunity at the site of infection and controls *B. pertussis* bacterial burden in the mouse upper and lower respiratory tract.

## Materials and methods

### Bacterial strains, media, and growth conditions

*B. pertussis* strain Bp536 (32) was maintained on BG agar (Difco) containing 10% defibrinated sheep’s blood (Hemostat, Dixon, CA) supplemented with 100 μg/ml streptomycin. For animal inoculations, liquid cultures were grown at 37°C on a roller drum to OD_600_ ≈ 1.0 in Stainer-Scholte medium supplemented with 1 mg/mL heptakis (Sigma) and 100 μg/ml streptomycin.

### Animals

All experiments were approved by the Ohio State University Institutional Animal Care and Use Committee under protocol number 2017A00000090-R1 and adhered to the NIH Guide for the Care and Use of Laboratory Animals. C57BL/6J mice were purchased from Jackson Labs and bred in-house. Male and female mice (6-12 weeks old) were used for all studies.

### Reagents

Boostrix (GSK) was purchased from the Ohio State University Medical Center Pharmacy. Purified filamentous hemagglutinin (FHA) from *B. pertussis* was purchased from List Biologicals (Campbell, CA) or over-produced in *E. coli*. Detoxified pertussis toxin (PT) purified from *B. pertussis* was purchased from List Biologicals. BcfA was over-produced in *E. coli* and purified as described previously (29) with endotoxin levels as reported in (30). Pertactin (PRN) was purchased from List Biologicals. Endotoxin in FHA and PT was <20 EU/mg. Endotoxin levels in all proteins were below that of Boostrix (33). GentleMACS lung dissociation kit was purchased from Miltenyi Biotec. RPMI, Ca^2+^/Mg^2+^-free PBS, ACK red blood cell lysis buffer, protein transport inhibitor, and fixation/permeabilization buffers were purchased from Life Technologies. Fetal bovine serum was purchased from Sigma-Aldrich (St. Louis, MO). U-Plex biomarker assay was purchased from Meso Scale Diagnostics (Rockville, MD). Antibodies and live/dead stains for flow cytometry were purchased from Life Technologies, BioLegend, or BD Biosciences.

### Immunizations

Mice were lightly anesthetized with 2.5% isoflurane/O_2_ for i.m. or i.n. immunization on day 0 and boost on day 28-35 (34) with the following vaccines: a) 1/5^th^ human dose of Boostrix (100 µL) delivered i.m. in the right deltoid and b) 1/5^th^ human dose of Boostrix with the addition of 10 μg BcfA (aPV/BcfA) incubated at room temperature for 30 minutes with rotation to adsorb the BcfA to alum, then resuspended in 100 µL of sterile PBS for i.m. injection or 50 uL sterile PBS for i.n. immunization, divided between both nares.

### Intravascular staining for discrimination between circulating and resident cells

Anti-CD45-PE (Clone 30-F11, BD Biosciences) (3 µg in 100 µL sterile PBS) was injected i.v. 10 minutes prior to sacrifice to label circulating lymphocytes, while resident lymphocytes were protected from labeling (35, 36). Peripheral blood was collected at time of sacrifice and checked by flow cytometry to confirm that >90% of circulating lymphocytes were CD45-PE+.

### Tissue dissociation and flow cytometry

**Figure 1:**
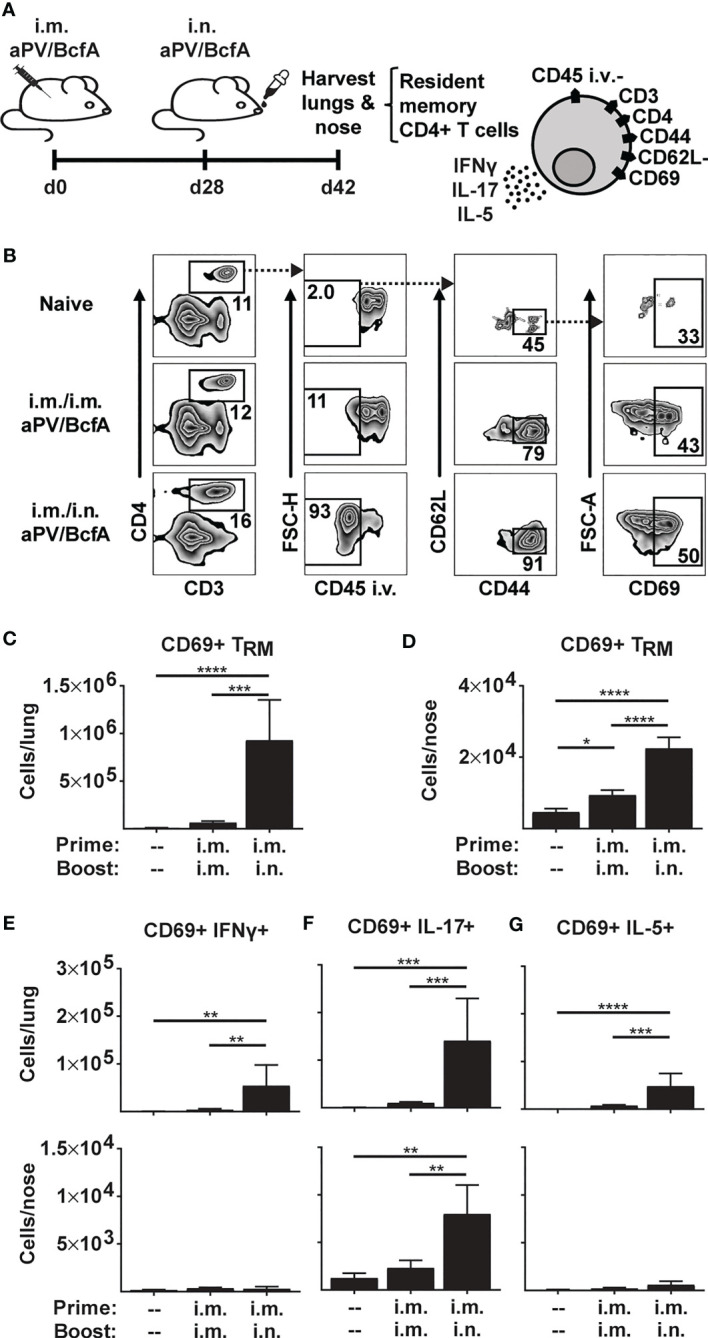
Prime-pull immunization with aPV/BcfA elicits IL-17-producing CD4+ T_RM_ in lungs and nose. **(A)** C57BL/6 mice (5/group) were immunized on d0 and d28 with 1/5 human dose aPV + 10μg BcfA (aPV/BcfA) delivered i.m./i.m. or i.m./i.n. T_RM_ in lungs and nose were quantified via flow cytometry on a BD Fortessa flow cytometer as live, CD3+, CD4+, CD45 i.v.-, CD44+, CD62L-, CD69+ cells. **(B)** Representative gating to identify T_RM_ in lungs. Number of CD69+ T_RM_ in **(C)** lungs and **(D)** nose. Number of CD69+ T_RM_ that were IFNγ+ **(E)**, IL-17+ **(F)**, or IL-5+ **(G)** following PMA/I stimulation. Data representative of two independent experiments and presented as mean +/- SEM. Data were analyzed by one-way ANOVA with Holm-Sidak correction for multiple comparisons. *P<0.05, **P<0.01, ***P<0.001, ****P<0.0001.

**Figure 2:**
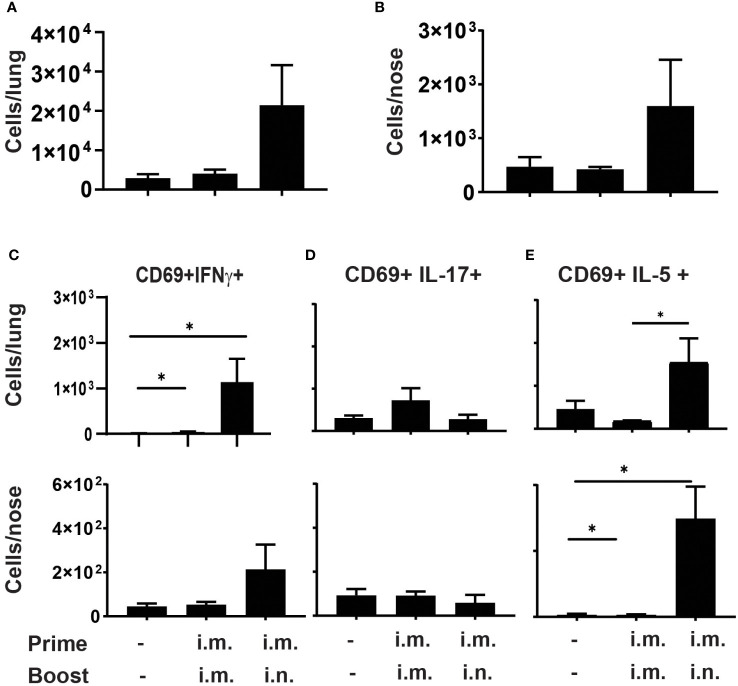
Prime-pull immunization with aPV elicits Th2 polarized CD4+ T_RM_ C57BL/6 mice (5/group) were immunized on d0 and d28 with 1/5 human dose aPV delivered i.m./i.m. or i.m./i.n. T_RM_ in lungs and nose were quantified via flow cytometry on a Cytek Aurora spectral flow cytometer as live, CD3+, CD4+, CD45 i.v.-, CD44+, CD62L-, CD69+ cells. Number of CD69+ T_RM_ in **(A)** lungs and **(B)** nose. Number of CD69+ T_RM_ in the lungs and nose that were IFNγ+ **(C)**, IL-17+ **(D)**, or IL-5+ **(E)** following PMA/I stimulation. Data representative of two independent experiments and presented as mean +/- SEM. Data were analyzed by one-way ANOVA with Holm-Sidak correction for multiple comparisons. *P<0.05.

Lung and nose tissues were enzymatically digested with GentleMACS lung dissociation kit (Miltenyi) according to the manufacturer’s instructions. Cell suspensions were filtered (40-μm) and red blood cells lysed with ACK lysis buffer. To detect cytokines, cells were stimulated with PMA (50 ng/ml) and ionomycin (500 ng/ml) in the presence of a protein transport inhibitor cocktail (eBioscience) for 4 h at 37°C. Cells were incubated with LIVE/DEAD Aqua or Near Infrared viability stains, followed by incubation with α-CD16/CD32 FcγRIII to block IgG Fc receptors. The following antibodies specific for cell surface markers were used: anti-CD3-V450 (clone 17A2), anti-CD4-BV750 (clone H129.19), anti-CD4 PE-CF594, anti-CD44-PerCP-Cy5.5 (clone IM-7), anti-CD62L-BV605 (clone MEL-14), anti-CD69-BV711 (clone HI.2F3), and anti-CD103-PE-CF594 (clone M290). For detection of cytokines, cells were fixed, permeabilized, and stained with antibodies against IFNγ (FITC conjugated clone XMG1.2), IL-17 (PE-Cy7 conjugated clone eBio17B7), and IL-5 (APC conjugated clone TRFK5). Fluorescence minus one or isotype control antibodies were used as negative controls. Samples were acquired on BD Fortessa (**Figure 1**) or Cytek Aurora (**Figure 2**) cytometers and data were analyzed with FlowJo software. The number of cells within each population were calculated by multiplying the frequency of live singlets in the population of interest by the total number of cells in each sample.

### Bacterial challenge

Bacterial strain Bp536 grown overnight to OD_600_ ≈ 1.0 was diluted in PBS to 5x10^5^ bacteria per 50 μl. On day 14 post-boost, mice were lightly anesthetized with 2.5% isoflurane/O_2_ and the 50 μl inoculum was divided between both nares as reported previously (34).

### Bacterial enumeration

Mice were sacrificed at 1-, 4-, 7-, and 14-days post challenge. Lungs and nasal tissue (including septum, turbinates, sinuses, and nasal associated lymphoid tissue (NALT)), were harvested, mechanically disrupted in PBS + 1% casein, and plated in serial dilutions on BG agar plates containing 10% sheep’s blood and supplemented with 100 μg/ml streptomycin. Colony forming units (CFUs) were counted after 4 days of incubation at 37° C. Data were transformed to log_10_.

### Antibody analysis

Serum from blood and supernatants from lung digestion were analyzed for the presence of antigen-specific antibodies. Purified antigens FHA, PT, and PRN were coupled through an amine linkage to MagPlex C magnetic microspheres (Luminex Corporation), each with a unique fluorescent bead region address, and combined to form a 5-plex microarray. Mouse serum or lung homogenates were diluted in assay buffer, PBS–0.1% Brij-35–1% bovine serum albumin (BSA), pH 7.2, and incubated with the beads for 2 h at room temperature (r.t.) in the dark while shaking at 800 rpm. After washing, appropriate biotinylated detection antibody was added, i.e., goat anti-mouse total IgG, rat anti-IgG1, rat anti-IgG2b, or goat anti-IgG2c, and rat anti-IgA at a 1:250 dilution in assay buffer for 1 h at r.t. After washing, streptavidin-phycoerythrin (SA-PE) at 1:250 in assay buffer was added for 1 h with shaking. Unbound SA-PE was removed by washing, and the beads were resuspended in 100 μl PBS prior to reading on a Luminex 200 flow cytometer. Antibody isotype and subclass values are reported in arbitrary luminescent intensity units (RLU).

## Results

### I.m. priming followed by i.n. booster with acellular vaccines elicits CD4+ T_RM_ in the nose and lungs of mice

Mucosal cellular and humoral adaptive immune responses against respiratory pathogens are critical to clear infections from the respiratory tract (37, 38) and studies in murine models showed that CD4+ T cells are necessary for clearing natural *B. pertussis* infections (39). Experimental pertussis vaccines, when delivered i.n., generate CD4+ T_RM_ cells in mice which prevent nasal colonization (25, 27). However, alum-adjuvanted aPV delivered i.m. alone do not generate CD4+ T_RM_ in the nose or lungs (1).

Here, we tested whether i.m. priming and i.n. booster with aPV/BcfA would elicit CD4+ T_RM_. We immunized C57BL/6 mice with aPV/BcfA via two different routes: i.m. prime and boost (i.m./i.m.), and i.m. prime followed by i.n. boost (i.m./i.n.). Mice were immunized on d0, boosted on d28, and the number and phenotype of CD4+ T cells in the nose and lungs were evaluated on d42 (2 weeks post-boost) (**Figure 1A**). Anti-CD45-PE antibody was injected i.v. 10 minutes before sacrifice and tissue harvest to distinguish circulating from tissue-resident memory cells (36). Single cell suspensions of lungs and noses were stimulated with PMA/Ionomycin and stained to identify the number of CD4+ T_RM_. The flow cytometry gating strategy is shown in **Figure 1B**. The number of live, CD3+, CD4+, and CD45 i.v.- cells that were CD44+CD62L-CD69+ (CD4+ T_RM_) were quantified. CD4+ T_RM_ cells were detected in the lungs (**Figure 1C**) and noses (**Figure 1D**) of i.m./i.n. immunized mice. However, little induction of T_RM_ in the tissues of i.m./i.m. immunized animals was observed. Lung T_RM_ of i.m./i.n.-vaccinated mice produced a small number of IFNγ+ cells (**Figure 1E**) that primarily produced IL-17 (**Figure 1F**) and few IL-5+ T_RM_ (**Figure 1G**)., while nose T_RM_ had negligible IFNγ- (**Figure 1E**) or IL-5- (**Figure 1G**) producing cells and exclusively produced IL-17 (**Figure 1F**). Thus, the addition of BcfA to aPV generated Th1 and Th17 polarized CD4+ T_RM_ that are correlated with protection against *B. pertussis* infection (40).

We then tested whether im./i.n. immunization with aPV alone would generate CD4+ T_RM_. C57BL/6 mice were immunized with aPV alone i.m./i.m. or i.m./i.n. on d0 and d28 and harvested on d42.Lungs and nose cells were stimulated with PMA/Ionomycin, followed by staining for CD4+ T_RM._ The gating strategy is shown in **Figure S1**. As observed with aPV/BcfA immunization, i.m./i.m. immunization with aPV alone did not elicit CD4+ T_RM_ in the lungs (**Figure 2A**) or nose (**Figure 2B**). I.m./i.n. immunization with aPV elicited CD4+ T_RM_ in the lungs (**Figure 2A**) and nose (**Figure 2B**). Intracellular cytokine staining showed that CD4+ T_RM_ from lungs and nose produced IFNγ (**Figure 2C**), no IL-17 (**Figure 2D**), and considerable IL-5 (**Figure 2E**), demonstrating the Th2 polarization of the mucosal T cell response in the nose and lungs in aPV-immunized mice. Thus, the same immunization regimen employed with aPV and aPV/BcfA elicits different CD4+ T_RM_ phenotypes.

### Prime-pull immunization generates mucosal and systemic antibodies

**Figure 3:**
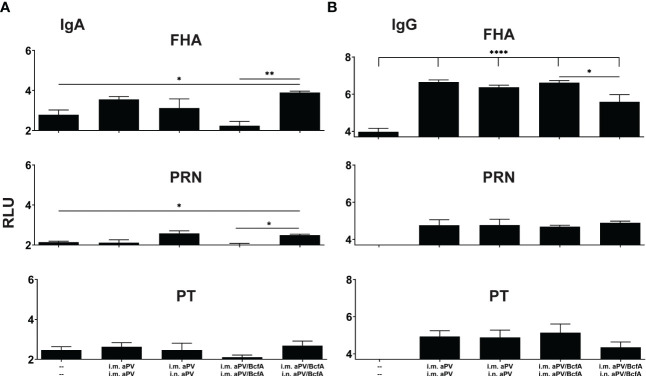
I.m/i.n immunization with aPV and aPV/BcfA generates mucosal and systemic IgA and IgG antibodies. Antibodies in lung homogenates and serum collected at 2 weeks post-boost (d42) were evaluated by an antigen-specific multiplex assay. FHA-, PRN-, and PT-specific IgA **(A)** and IgG **(B)** in the lungs. Relative luminescence units were log_10_ transformed. Data are presented as mean +/- SEM with samples from 5-6 mice/group. One representative experiment of two independent experiments is shown. Data were analyzed by one-way ANOVA with Holm-Sidak correction for multiple comparisons. *P<0.05, **P<0.01, ****P<0.0001.

Natural infection or i.n. immunization with experimental pertussis vaccines generates both IgG and IgA responses in the lungs (27, 41, 42), which are important for immunity against several respiratory pathogens (37). We quantified antigen-specific IgA and IgG in the lungs of mice immunized with aPV or aPV/BcfA delivered i.m./i.m. or i.m./i.n. aPV delivered by i.m./i.m. or i.m./i.n. immunization did not elicit IgA responses in the lungs against the background of unimmunized mice (**Figure 3A**). Mice immunized with aPV/BcfA through the i.m./i.n. route elicited higher FHA and PRN specific IgA antibodies in the lungs compared with naïve mice and mice immunized i.m./i.m. with aPV/BcfA (**Figure 3A**). However, there was no significant difference in IgA levels between aPV- and aPV/BcfA- immunized mice. Serum IgA was not increased compared to naïve mice with either the vaccine or immunization route (data not shown).

FHA-specific IgG levels in the lungs were increased with both aPV and aPV/BcfA immunization compared to naïve mice (**Figure 3B**). There was a slight decrease in FHA-specific IgG in the lungs of mice immunized with i.m./i.n. aPV/BcfA compared to i.m./i.m. delivery of the same vaccine. PRN and PT specific IgG were not increased above naïve mice. FHA, PRN, and PT specific serum IgG levels in immunized mice were higher than in naïve mice. Anti-PT serum IgG levels were slightly lower in mice immunized i.m./i.n. with aPV/BcfA, compared to mice immunized i.m./i.m. with the same formulation (data not shown).

**Figure 4:**
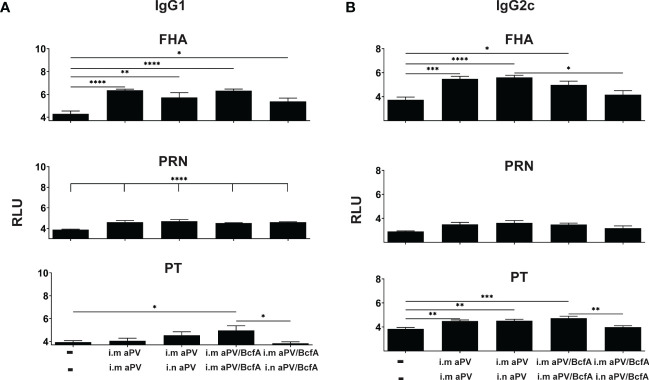
Lung IgG isotypes are similar in aPV and aPV/BcfA immunized mice. IgG1 **(A)** IgG2c **(B)** antibody isotypes in lung homogenate were quantified by multiplex assay in samples shown in **Figure 3**. Data were analyzed by one-way ANOVA with Holm-Sidak correction for multiple comparisons. *P<0.05, **P<0.01, ***P<0.001, ****P<0.0001.

We then quantified the IgG isotypes in the lungs (**Figure 4**). FHA and PRN-specific IgG1 was higher in vaccinated groups compared to naïve animals but did not differ between vaccines. Anti-PT antibodies were increased in the lungs following i.m./i.m. immunization with aPV/BcfA. Interestingly, compared to naïve mice, anti-PT responses did not increase in the lungs of mice immunized i.m/i/n with aPV/BcfA. Similarly, anti-FHA and anti-PT IgG2c was elevated by immunization in all groups except in mice immunized i.m./i.n.with aPV/BcfA (**Figure 4B**). In this group, anti-FHA and anti-PT IgG2c was lower in mice immunized i.m./i.n. with aPV/BcfA compared to mice immunized i.m./i.m. with aPV/BcfA. IgG2b did not increase following immunization (data not shown). Overall, these data suggest that i.m./i.n. immunization with aPV/BcfA does not increase antigen-specific IgG responses in the lungs compared to i.m./i.m. immunization with aPV/BcfA or compared to aPV alone delivered i.m./i.m. or i.m./i.n. While IgG isotypes in the serum in all immunization groups were also increased compared to naïve mice (**Figure S2**), there was little difference in the isotypes between immunization groups. Overall, prime-pull immunization with either aPV or aPV/BcfA did not increase total IgG and IgG isotypes in the serum and lungs compared to systemic prime and boost alone.

### Prime-pull immunization with aPV/BcfA reduces *B. pertussis* nasal colonization

**Figure 5:**
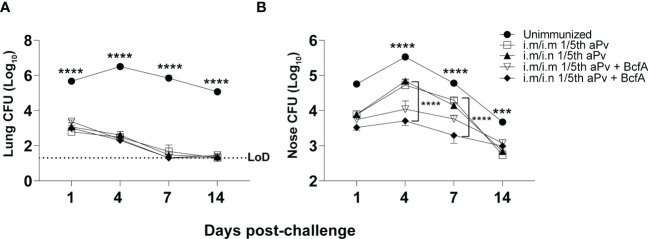
Prime-pull immunization with aPV/BcfA reduces *B. pertussis* burden in the nose upon challenge. C57BL/6 mice were primed on d0 and boosted on d28 with the indicated vaccines and immunization routes. Unimmunized and immunized mice were challenged i.n. on d42 with 5x10^5^ CFU Bp536. **(A)** Lungs and **(B)** nose were harvested at the indicated time points and CFUs enumerated on BG agar plates. 5 mice/group. Dotted line represents limit of detection (LOD) for lung at 20 CFU. LOD for nose is 30 CFU (below the scale). Data were analyzed by two-way ANOVA with Holm-Sidak correction for multiple comparisons. Black asterisks indicate significant differences between unimmunized mice and the immunization groups at all time points post-challenge. The brackets indicate statistical differences between i.m./i.m. aPV and i.m./i.n. aPV/BcfA at day 4 and day 7 post-challenge. ***P<0.001, ****P<0.0001 by ANOVA.

We then measured the effect of systemic priming and mucosal booster on *B. pertussis* respiratory tract colonization. Mice primed i.m. with aPV alone or aPV/BcfA were boosted i.m. or i.n. with the same formulation. Two weeks post-boost mice were i.n. challenged with 5x10^5^ CFUs of *B. pertussis* strain Bp536. Lungs and nasal tissue were harvested on days 1-14 post-challenge for enumeration of CFUs. Bacterial burden in the lungs was reduced by more than 2 logs in all immunized mice compared to naïve mice by 1dpi. All lungs of immunized mice had bacterial burden below the limit of detection by 14 dpi while naïve challenged mice remained colonized (**Figure 5A**). Bacterial burden in the nose was reduced on d4 and d7 post-challenge in mice immunized i.m/i.n. with aPV/BcfA (**Figure 5B**), compared to mice immunized i.m/i.n. with aPV alone or in unimmunized challenged mice. Interestingly, there was a slight but significant reduction in nasal colonization of mice immunized i.m/i.m with aPV/BcfA compared to mice immunized i.m/i.m with aPV alone (**Figure 5B** and **Figure S3**) or naïve mice. (Comparisons between vaccine formulations and immunization routes are separated for clarity in **Figure S3**). Thus, the immune response elicited by the addition of BcfA to aPV was more effective than aPV alone in reducing *B. pertussis* colonization of the nose.

## Discussion

Current aPV fail to prevent nasal colonization of *B. pertussis* and may, in fact, exacerbate nasal colonization (2, 23). Thus, the large cohort of people who have been immunized i.m. with aPV may carry *B. pertussis* asymptomatically and transmit the infection to infants and other vulnerable populations. This may be due to the immunization route or the Th2 polarized immune responses elicited by alum. Here, we tested whether combining systemic priming with an i.n. booster for a “prime-pull” strategy would elicit CD4+ T_RM_ in the upper and lower respiratory tracts, and whether the addition of BcfA to aPV would change the T-helper phenotype of the resulting T_RM_ cells. Prime-pull immunization with aPV or aPV/BcfA elicited CD4^+^ T_RM_, showing that mucosal booster immunization is capable of recruiting T cells to the lungs and nose of mice, regardless of formulation. We previously showed that i.m. immunization of mice with aPV/BcfA elicited Th1/17 systemic T cell responses compared to Th1/2 systemic responses elicited by aPV alone (30). We found a similar trend when comparing the T-helper phenotype of mucosal T_RM_ between vaccine compositions. I.m./i.n. administration of aPV generated IFNγ+ and IL-5+ T_RM_ in the lungs, and primarily generated IL-5+ T_RM_ in the nose, demonstrating a Th2-polarized response. In contrast, i.m./i.n. administration of aPV/BcfA generated IFNγ+, IL-5+, and IL-17+ T_RM_ in the lungs, but exclusively IL-17+ T_RM_ in the nose, demonstrating a Th1/17-polarized response. Notably, IL-5+ T_RM_ were not detected in the nose of i.m./i.n. aPV/BcfA-immunized mice. Thus, adding BcfA to aPV changed the mucosal T cell responses from a Th2 polarized to a Th17 polarized phenotype. This result suggests that the phenotype of the T_RM_ is shaped by the properties of the adjuvant combination.

Systemic immunization with aPV alone does not elicit mucosal antibodies (1, 23, 27). We show that PT-specific IgA was elicited in the lungs of mice that received prime-pull immunization with either aPV or aPV/BcfA, but not i.m. delivery alone. FHA-specific IgA was elicited in the lungs by i.m./i.n. immunization with aPV/BcfA, but not aPV alone, suggesting that adding BcfA to aPV elicits IgA in the mucosa. IgG was elicited in the lungs and serum of mice immunized with both aPV and aPV/BcfA, with a slight reduction in total IgG and some IgG isotypes when aPV/BcfA was delivered i.m./i.n. These data suggest that the composition of mucosal antibodies is determined by the immunization route and the properties of the adjuvant.

We showed previously that i.m. immunization of mice with aPV/BcfA accelerates bacterial clearance of *B. pertussis* from murine lungs compared to i.m. aPV (30). Here we show that lung colonization is reduced compared to unimmunized mice with both vaccine compositions and immunization routes. In the previous study, aPV/BcfA included a higher dose of BcfA than was used in the current work, which allowed us to discern differences following immunization with 1/5^th^ human dose aPV. Notably, in this study, CFUs in the nose were significantly reduced on day 4 and day 7 in mice immunized i.m./i.n. with aPV/BcfA, compared to unimmunized mice and mice immunized with aPV alone, suggesting that immune responses elicited by aPV/BcfA are more effective in the upper respiratory tract.

These data have several implications. The adjuvant composition of the vaccine determines the T-helper phenotype of the immune response, regardless of immunization route. The observation that i.m./i.n. immunization with aPV/BcfA but not aPV alone reduced *B. pertussis* CFUs from the nose implies that the Th17 polarized T_RM_ elicited by aPV/BcfA are important for protection of the upper respiratory tract. The systemic and mucosal antibody response generated by aPV and aPV/BcfA was similar, suggesting that the T cells have a greater contribution than antibodies to bacterial clearance from the upper respiratory tract. This conclusion is supported by previous studies in mice which established that CD4^+^IL-17^+^ T_RM_ are critical for clearance of *B. pertussis* from the nose (1). Splenic T cell responses generated by prime-pull immunization with aPV alone were also Th2 polarized, while the splenic T cell responses generated by aPV/BcfA were Th1/Th17 polarized (data not shown). These data also show that the mucosal immune phenotype elicited by alum-adjuvanted aPV can be modified by the addition of BcfA, and imply that modification of the Th2-polarized aPV-primed response to a more Th17-polarized response may improve protection of the upper respiratory tract. In this study we used 1/5^th^ human dose of aPV for mouse immunizations, while other groups deliver as little as 1/100^th^ human dose of aPV (1, 43). Despite this high vaccine dose, the addition of BcfA modified the aPV immune response and reduced nasal colonization. We are testing lower aPV vaccine doses which may provide a larger window of time to observe differences elicited by the addition of BcfA to the vaccine. Overall, this work suggests that immunization of unimmunized individuals using an i.m. priming and i.n. booster regimen with a BcfA-adjuvanted vaccine may be effective in preventing establishment of a *B. Pertussis* nasopharyngeal reservoir and thereby reduce *B. pertussis* transmission in humans.

## Data availability statement

The raw data supporting the conclusions of this article will be made available by the authors, without undue reservation.

## Ethics statement

The animal study was reviewed and approved by Ohio State University Institutional Animal Care and Use Committee.

## Author contributions

KY, JH, KC, MS, MG, KM, and AF conducted mouse experiments, SQ conducted multiplex antibody analysis, YH and JM produced FHA protein, KY, JH, PD, and RD designed experiments, KY, JH, MS, and PD analyzed data, and KY, JH, and PD wrote the manuscript. All authors contributed to the article and approved the submitted version.

## Supplementary material

Supplementary Figure 1 Gating strategy to identify CD4+ T_RM_ in the nose and lungs of aPV immunized mice. Lung and nose tissues were enzymatically digested and stained with antibodies and collected on a Cytek Aurora spectral flow cytometry. T_RM_ were identified as live, CD3+, CD4+, CD45 i.v.-, CD44+, CD62L-, CD69+ cells.

Supplementary Figure 2 Similar systemic IgG responses are elicited by aPV and aPV/BcfA. IgG1 and IgG2c antibody isotypes in serum were quantified by multiplex assay in samples shown in . Data were analyzed by one-way ANOVA with Holm-Sidak correction for multiple comparisons. *P<0.05, **P<0.01, ***P<0.001, ****P<0.0001.

Supplementary Figure 3 aPV/BcfA immunization reduces *B. pertussis* nasal colonization. Data in were separated according to immunization regimen and vaccine type. **(A)** Mice immunized i.m./i.m. with aPV alone or aPV/BcfA; **(B)** Mice immunized i.m./i.n. with aPV or aPV/BcfA. **(C)** Mice immunized i.m./i.m. or i.m./i.n. with aPV alone **(D)** Mice immunized i.m./i.m. or i.m./i.n. with aPV/BcfA. *P<0.05, **P<0.01, ****P<0.0001. Black asterisks indicate significant differences between unimmunized mice and the immunization groups at each time point. The bracket indicates the differences between the indicated immunization groups at day 4 and day 7 post-challenge.
