# Supplementary figures and images for: Systemic priming and intranasal booster with a BcfA-adjuvanted acellular pertussis vaccine generates CD4+ IL-17+ nasal tissue resident T cells and reduces B. pertussis nasal colonization
Source: Front Immunol. 2023 May 18;14:1181876. doi: 10.3389/fimmu.2023.1181876 (PMC10232778; doi:10.3389/fimmu.2023.1181876)

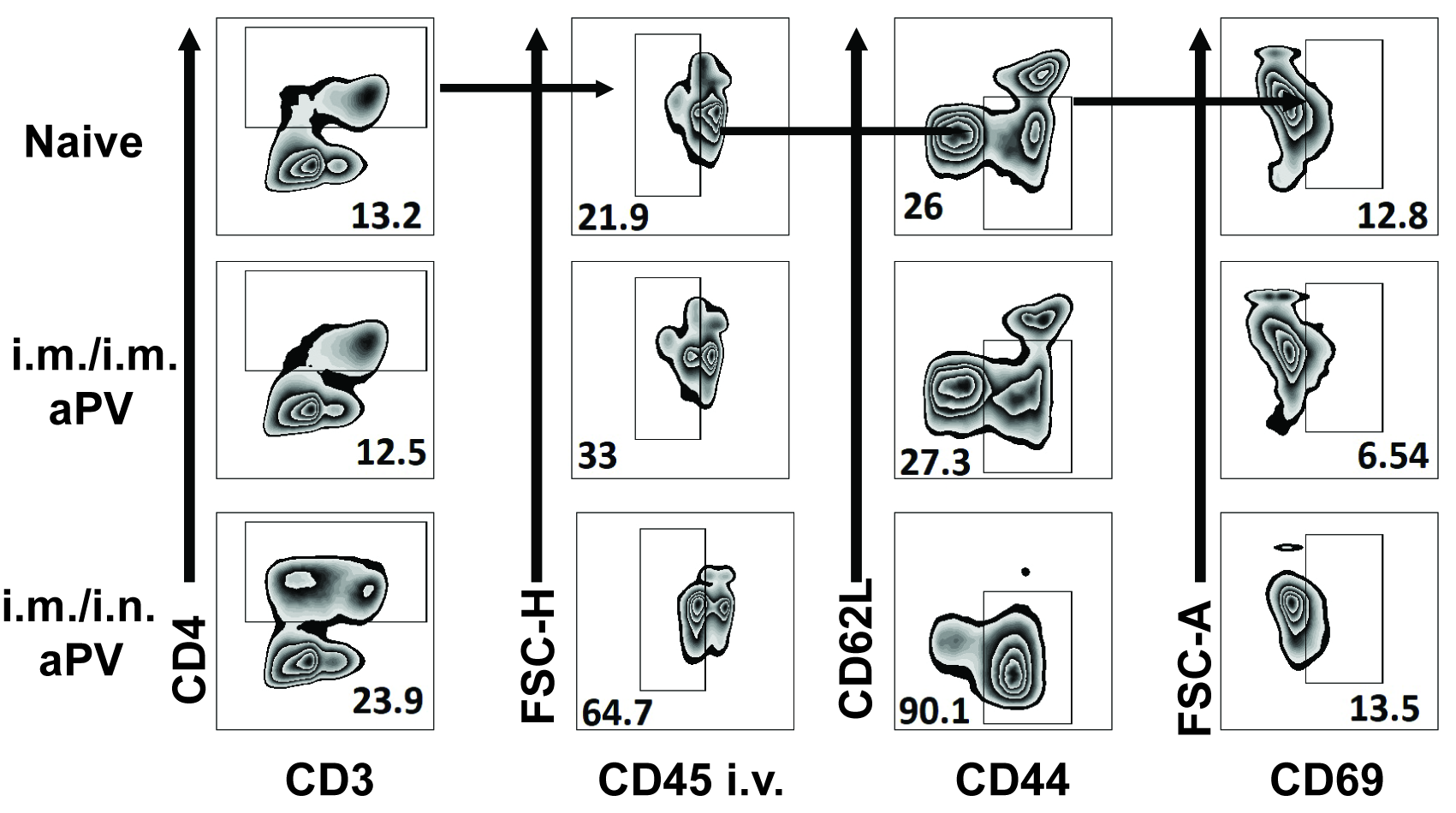

Supplement: Supplementary Figure 1 — Gating strategy to identify CD4+ TRM in the nose and lungs of aPV immunized mice. Lung and nose tissues were enzymatically digested and stained with antibodies and collected on a Cytek Aurora spectral flow cytometry. TRM were identified as live, CD3+, CD4+, CD45 i.v.-, CD44+, CD62L-, CD69+ cells. [file Image_1.tif]

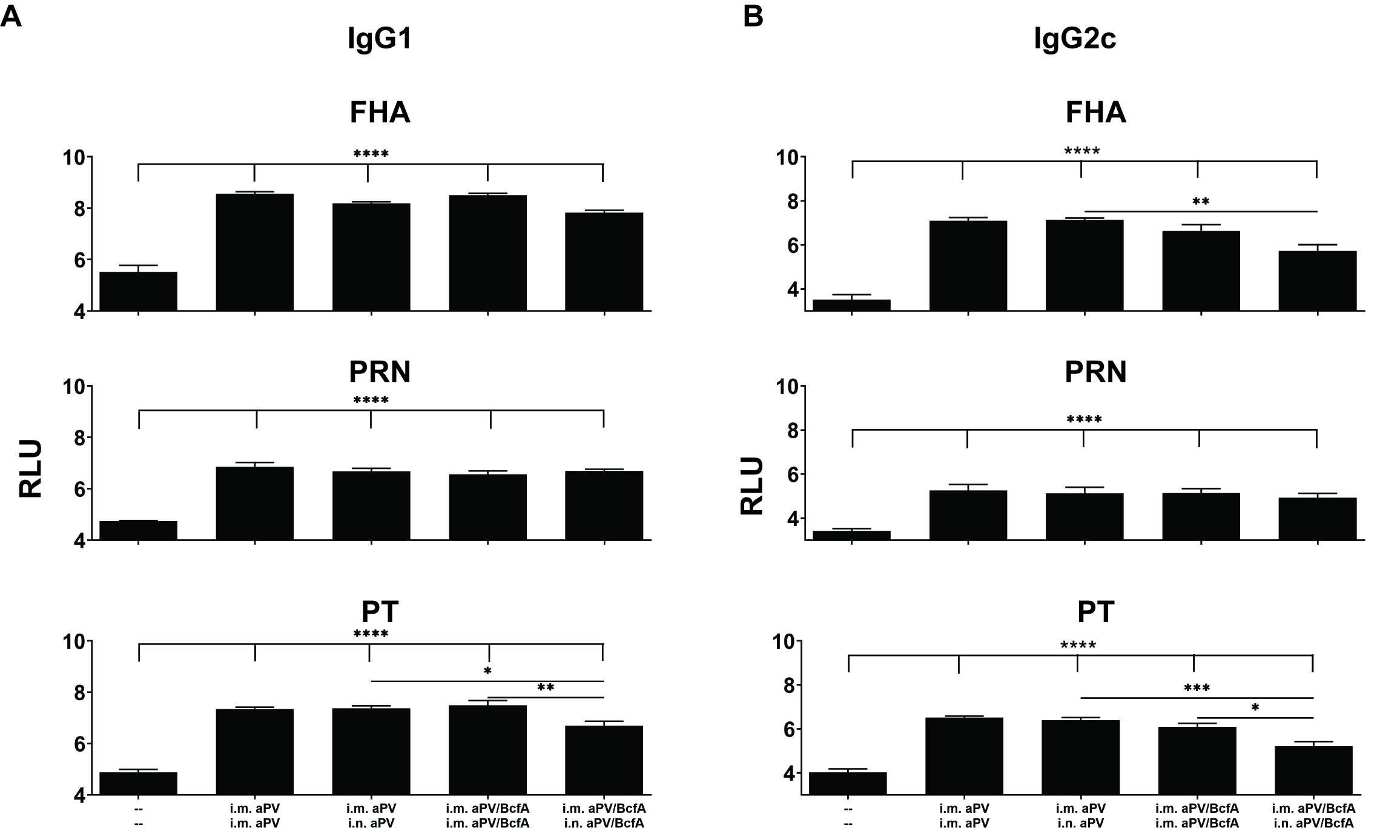

Supplement: Supplementary Figure 2 — Similar systemic IgG responses are elicited by aPV and aPV/BcfA. IgG1 and IgG2c antibody isotypes in serum were quantified by multiplex assay in samples shown in . Data were analyzed by one-way ANOVA with Holm-Sidak correction for multiple comparisons. *P<0.05, **P<0.01, ***P<0.001, ****P<0.0001. [file Image_2.tif]

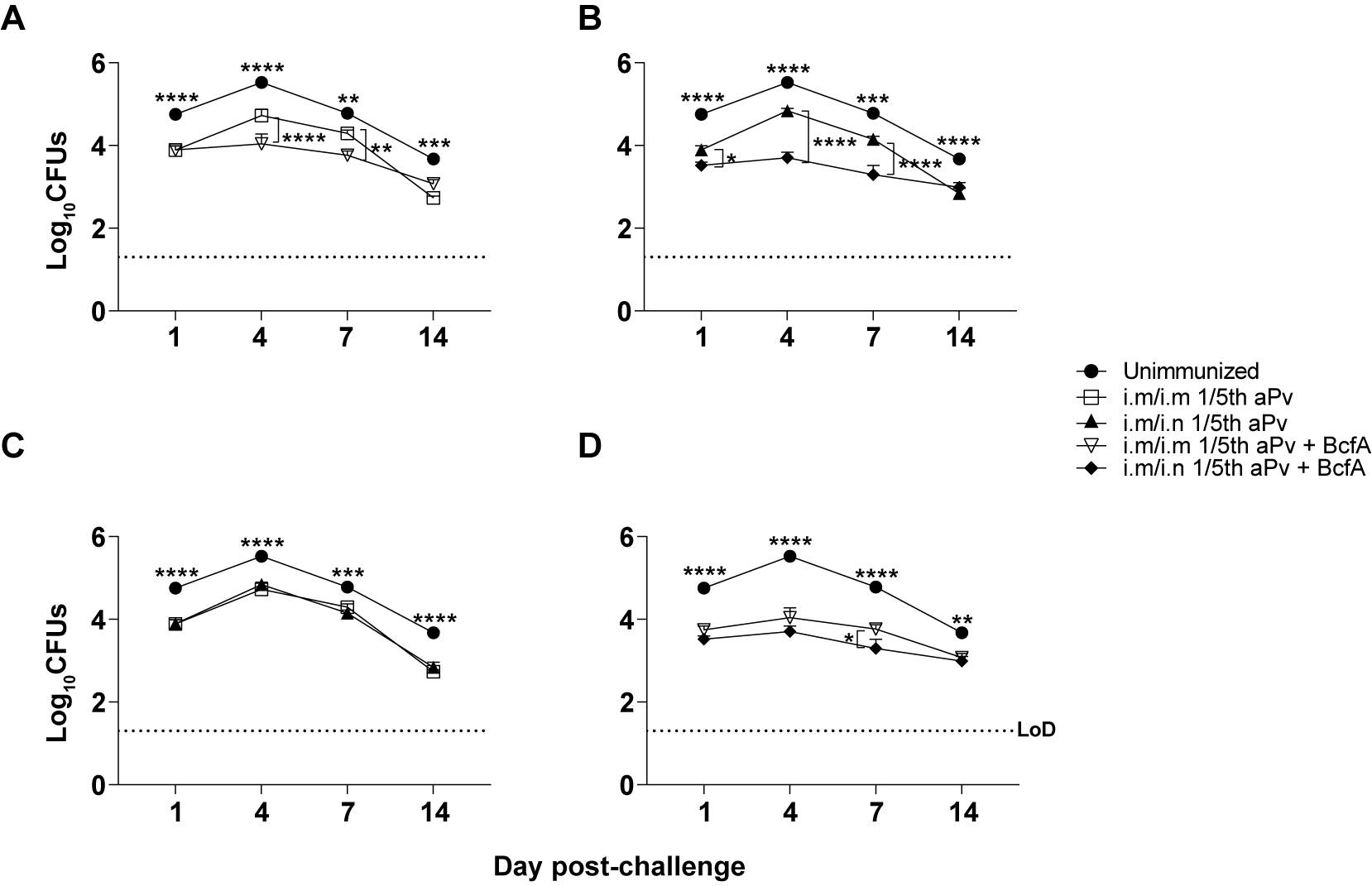

Supplement: Supplementary Figure 3 — aPV/BcfA immunization reduces B. pertussis nasal colonization. Data in were separated according to immunization regimen and vaccine type. (A) Mice immunized i.m./i.m. with aPV alone or aPV/BcfA; (B) Mice immunized i.m./i.n. with aPV or aPV/BcfA. (C) Mice immunized i.m./i.m. or i.m./i.n. with aPV alone (D) Mice immunized i.m./i.m. or i.m./i.n. with aPV/BcfA. *P<0.05, **P<0.01, ****P<0.0001. Black asterisks indicate significant differences between unimmunized mice and the immunization groups at each time point. The bracket indicates the differences between the indicated immunization groups at day 4 and day 7 post-challenge. [file Image_3.tif]
